# Immune cell profiles in the tumor microenvironment of early-onset, intermediate-onset, and later-onset colorectal cancer

**DOI:** 10.1007/s00262-021-03056-6

**Published:** 2021-09-16

**Authors:** Tomotaka Ugai, Juha P. Väyrynen, Mai Chan Lau, Jennifer Borowsky, Naohiko Akimoto, Sara A. Väyrynen, Melissa Zhao, Rong Zhong, Koichiro Haruki, Andressa Dias Costa, Kenji Fujiyoshi, Kota Arima, Kana Wu, Andrew T. Chan, Yin Cao, Mingyang Song, Charles S. Fuchs, Molin Wang, Jochen K. Lennerz, Kimmie Ng, Jeffrey A. Meyerhardt, Marios Giannakis, Jonathan A. Nowak, Shuji Ogino

**Affiliations:** 1 Program in MPE Molecular Pathological Epidemiology, Department of Pathology, Brigham and Women’s Hospital, and Harvard Medical School, Boston, MA, USA; 2 Department of Epidemiology, Harvard T.H. Chan School of Public Health, Boston, MA, USA; 3 Department of Medical Oncology, Dana-Farber Cancer Institute and Harvard Medical School, Boston, MA, USA; 4 Cancer and Translational Medicine Research Unit, Medical Research Center Oulu, Oulu University Hospital, and University of Oulu, Oulu, Finland; 5 Conjoint Gastroenterology Department, QIMR Berghofer Medical Research Institute, Queensland, Australia; 6 Department of Nutrition, Harvard T.H. Chan School of Public Health, Boston, MA, USA; 7 Channing Division of Network Medicine, Department of Medicine, Brigham and Women’s Hospital, Harvard Medical School, Boston, MA, USA; 8 Clinical and Translational Epidemiology Unit, Massachusetts General Hospital and Harvard Medical School, Boston, MA, USA; 9 Division of Gastroenterology, Massachusetts General Hospital, Boston, MA, USA; 10 Department of Immunology and Infectious Diseases, Harvard T.H. Chan School of Public Health, Boston, MA; 11 Division of Public Health Sciences, Department of Surgery, Washington University in St. Louis, St. Louis, MO, USA; 12 Alvin J. Siteman Cancer Center, Washington University School of Medicine, St. Louis, MO, USA; 13 Division of Gastroenterology, Department of Medicine, Washington University School of Medicine, St. Louis, MO, USA; 14 Yale Cancer Center, New Haven, CT, USA; 15 Department of Medicine, Yale School of Medicine, New Haven, CT, USA; 16 Smilow Cancer Hospital, New Haven, CT, USA; 17 Genentech, South San Francisco, CA, USA; 18 Department of Biostatistics, Harvard T.H. Chan School of Public Health, Boston, MA, USA; 19 Department of Pathology, Massachusetts General Hospital/Harvard Medical School, Boston, MA, USA; 20 Broad Institute of MIT and Harvard, Cambridge, MA, USA; 21 Department of Medicine, Brigham and Women’s Hospital and Harvard Medical School, Boston, MA, USA; 22 Cancer Immunology and Cancer Epidemiology Programs, Dana-Farber Harvard Cancer Center; Boston, MA, USA

**Keywords:** immunology, young-onset, colorectal neoplasms, molecular pathological epidemiology

## Abstract

**Background:**

Despite heightened interest in early-onset colorectal cancer (CRC) diagnosed before age 50, little is known on immune cell profiles of early-onset CRC. It also remains to be studied whether CRCs diagnosed at or shortly after age 50 are similar to early-onset CRC. We therefore hypothesized that immune cell infiltrates in CRC tissue might show differential heterogeneity patterns between three age groups (<50 “early-onset”, 50-54 “intermediate-onset”, ≥55 “later-onset”).

**Methods:**

We examined 1,518 incident CRC cases with available tissue data, including 35 early-onset and 73 intermediate-onset cases. To identify immune cells in tumor intraepithelial and stromal areas, we developed three multiplexed immunofluorescence assays combined with digital image analyses and machine learning algorithms, with the following markers: (1) CD3, CD4, CD8, CD45RO (PTPRC), FOXP3 for T cells; (2) CD68, CD86, IRF5, MAF, MRC1 (CD206) for macrophages; (3) ARG1, CD14, CD15, CD33, HLA-DR for myeloid cells.

**Results:**

Although no comparisons between age groups showed statistically significant differences at the stringent two-sided α level of 0.005, compared to later-onset CRC, early-onset CRC tended to show lower levels of tumor-infiltrating lymphocytes (P=0.013), intratumoral periglandular reaction (P=0.025), and peritumoral lymphocytic reaction (P=0.044). Compared to later-onset CRC, intermediate-onset CRC tended to show lower densities of overall macrophages (P=0.050), M1-like macrophages (P=0.062), CD14^+^HLA-DR^+^ cells (P=0.015), and CD3^+^CD4^+^FOXP3^+^ cells (P=0.039).

**Conclusions:**

This hypothesis-generating study suggests possible differences in histopathologic lymphocytic reaction patterns, macrophages, and regulatory T-cells in the tumor microenvironment by age at diagnosis.

## INTRODUCTION

Colorectal cancer (CRC) is a group of heterogeneous tumors with complex interactions between neoplastic and immune cells such as lymphocytes and tumor-associated macrophages in the tumor microenvironment (1–3). Evidence indicates influences of tumor molecular features such as microsatellite instability (MSI) status on immune reactions to tumor. A strong adaptive immune response enriched with cytotoxic and memory T-cells in tumor tissue has been associated with better survival in CRC patients (4–6), while the abundance of macrophages has also been associated with clinical outcome (7). In addition, myeloid-derived suppressor cells (MDSCs) are considered to contribute to cancer immune evasion (8). Improved understanding of tumor immune microenvironment will help advance immune-based cancer prevention and treatment strategies.

The incidence of early-onset CRC diagnosed before 50 years of age has been increasing in many parts of the world since the 1980s (9, 10). The rise in early-onset cancers in various organs (including the colorectum) has been a growing global concern (11) and a topic of the top 2020 Provocative Question of the USA National Cancer Institute. Previous studies have shown heterogeneity of tumor molecular characteristics between early-onset CRC and CRC diagnosed at age ≥50 and within early-onset CRCs (12–20). This suggests possible differences in immune cell infiltrates between early-onset and CRC diagnosed at age ≥50. However, it is unclear whether there may or may not exist a sharp dichotomy in features of CRC at age 50. We speculated a possibility of an “age continuum” in features of CRC (9).

To clarify this issue, we examined characteristics of early-onset CRC as well as CRC diagnosed at age 50-54 (hereafter referred to as “intermediate-onset CRC”). We tested the hypothesis that profiles of immune cell infiltrates in tumor tissue might differ between three age groups (<50 “early-onset”, 50-54 “intermediate-onset”, ≥55 “later-onset”). We utilized a CRC database that included histopathological lymphocytic reaction patterns as well as the densities of T-cell, macrophage, and other myeloid cell populations assessed by multiplex immunofluorescence assays combined with digital image analyses and machine learning algorithms.

## METHODS

### Study population

**Figure 1.**
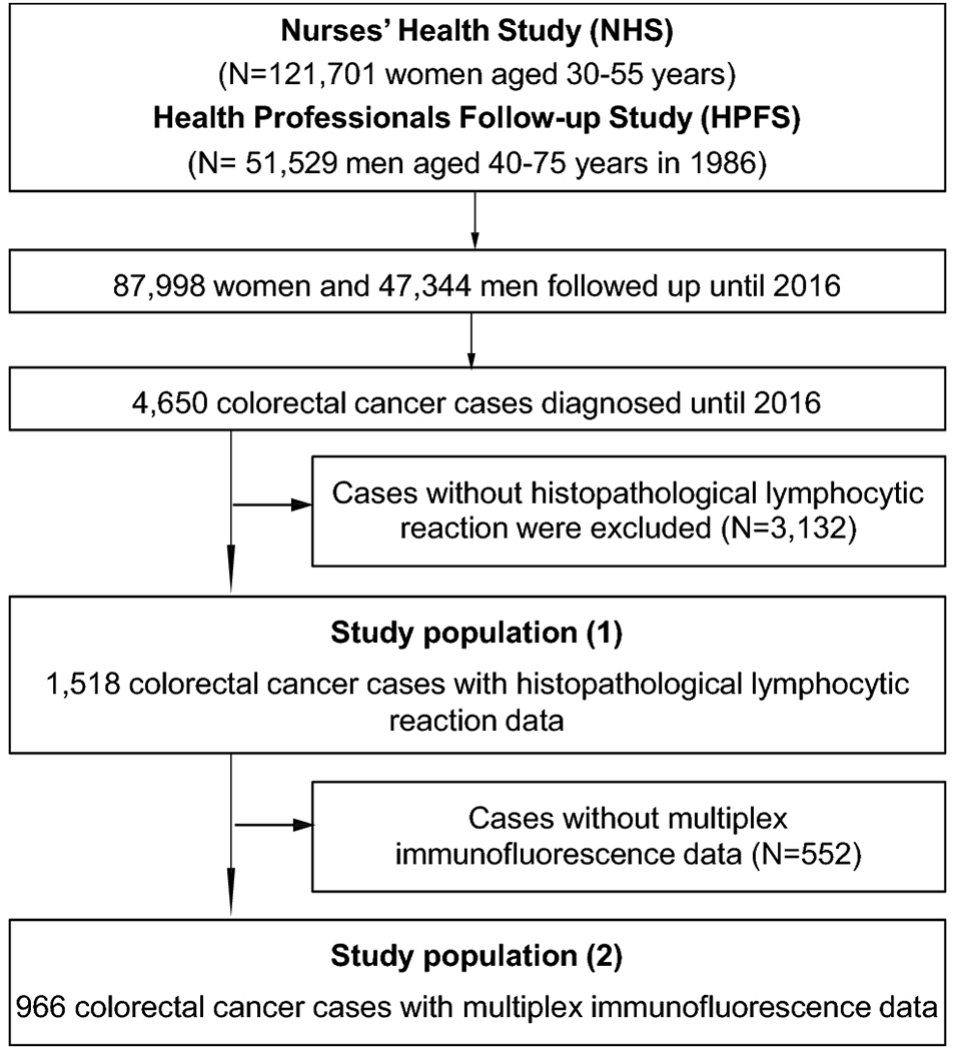
Flow diagram of study population in the Nurses’ Health Study and the Health Professionals Follow-up Study

We used a molecular pathological epidemiology database of 1,518 incident CRC cases with available tissue data, including 35 early-onset cases and 73 intermediate-onset cases, that had occurred in two U.S.-wide prospective cohort studies, namely the Nurses’ Health Study (NHS, 121,701 women aged 30-55 years at enrollment, followed since 1976) and the Health Professionals Follow-up Study (HPFS, 51,529 men aged 40 to 75 years at enrollment, followed since 1986) (Figure 1) (21). We included both colon and rectal cancer cases based on the colorectal continuum theory: i.e., a gradual change of clinical and tumor characteristics throughout the colorectum (22). Study participants in NHS and HPFS have been sent questionnaires biennially to update information on their lifestyle and newly-diagnosed diseases including incident CRC. The National Death Index was used to identify unreported lethal CRC cases. Study physicians reviewed medical records of CRC cases, confirmed the diagnosis, and collected data on tumor size, tumor anatomical location, and disease stage.

Informed consent was obtained from all study participants. The study protocol was approved by the institutional review boards of the Brigham and Women’s Hospital and Harvard T.H. Chan School of Public Health, and those of participating registries as required. We also obtained signed consents from patients (or next-of-kin, if patients died) to use tissue specimens.

### Tumor tissue analyses including immune cell assessments

We obtained formalin-fixed paraffin-embedded (FFPE) tumor tissue samples from hospitals throughout the U.S. where CRC patients underwent surgical resection. A single pathologist (S.O.), blinded to other data, performed a centralized review of hematoxylin and eosin-stained tissue sections from all CRC cases. Tumor differentiation was categorized as well to moderate vs. poor (> 50% vs. ≤ 50% glandular area, respectively). Four components of lymphocytic reaction to tumors, including tumor-infiltrating lymphocytes, intratumoral periglandular reaction, peritumoral lymphocytic reaction, and Crohn’s-like lymphoid reaction were graded as absent/low, intermediate, and high, as previously described (23, 24).

**Figure 2.**
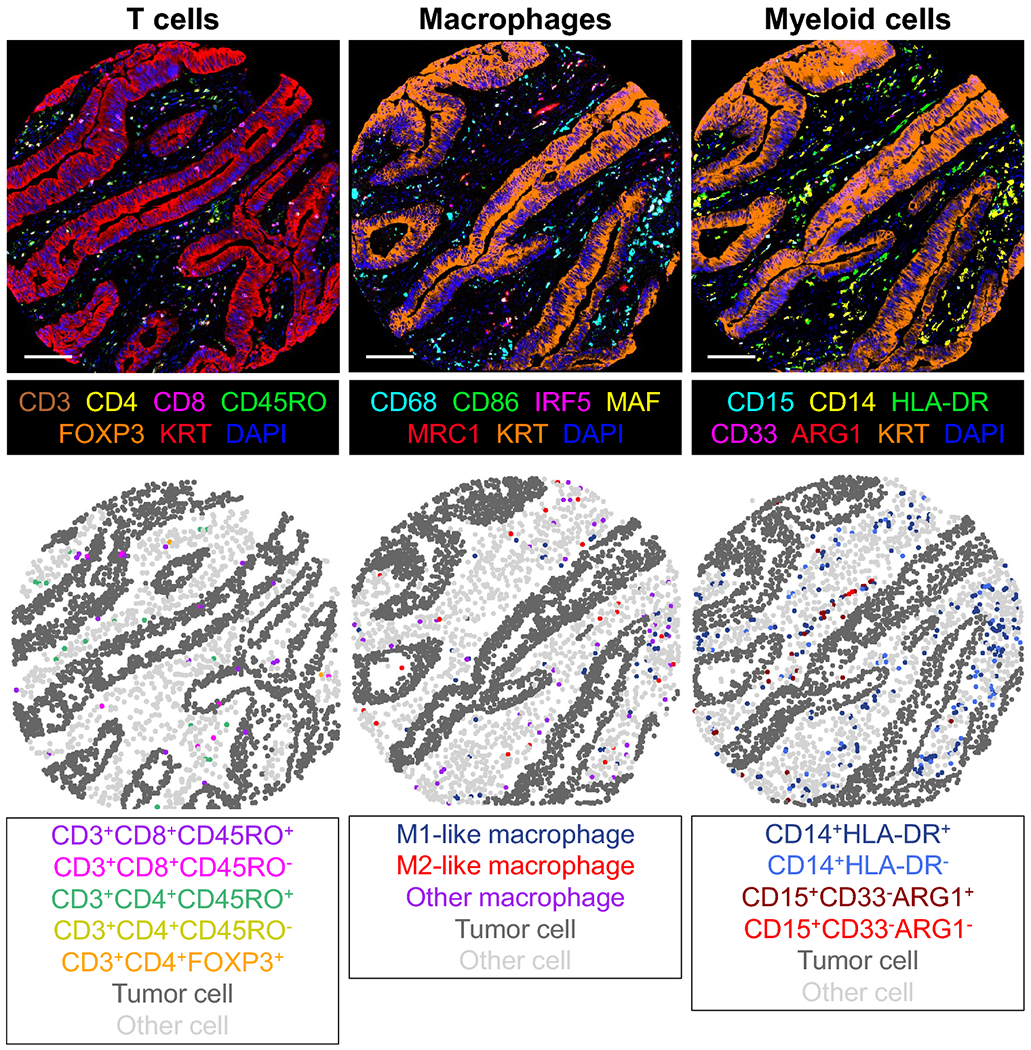
Representative images of immune profiling of a colorectal adenocarcinoma using three multiplex immunofluorescence assays combined with digital image analysis. The scale bars are 100 μm

Details of multiplex immunofluorescent assays have been described previously (25–28). Briefly, we constructed tissue microarrays consisting of up to four tumor cores from each case. We developed three assays of multiplexed immunofluorescence with the following markers: (1) CD3, CD4, CD8, CD45RO (one isoform of PTPRC gene products), and FOXP3 for T cells, (2) CD68, CD86, IRF5, MAF, and MRC1 (CD206) for macrophages, and (3) ARG1, CD14, CD15, CD33, and HLA-DR for myeloid cells (Figure 2) [following protein nomenclature recommendations by an expert panel (29)]. We obtained digital images at 200x magnification using an automated multispectral imaging system (Vectra 3.0, Akoya Biosciences, Hopkinton, MA). Using machine learning algorithm with pathologist’s supervision (Inform 2.4.1, Akoya Biosciences), immune cell densities (cells/mm^2^) in tumor intraepithelial and stromal areas were calculated through the process of tissue segmentation (classifying tissue regions into tumor epithelium, stroma, and other), cell segmentation (detecting cells and their nuclear, cytoplasmic, and membranous compartments), and cell phenotyping (classifying cells based on cell phenotypic features including fluorophore intensities and cytomorphology). We used immune cell densities in overall tumor region (tumor intraepithelial and stromal areas combined) in our primary hypothesis testing.

Genomic DNA was extracted from FFPE tissue blocks. Microsatellite instability (MSI) status was determined by 10 microsatellite markers (D2S123, D5S346, D17S250, BAT25, BAT26, BAT40, D18S55, D18S56, D18S67, and D18S487). High-level microsatellite instability (MSI) was defined as the presence of instability in ≥ 30% of the markers (22, 30). CpG island methylator phenotype (CIMP)-high was defined as ≥ 6 methylated promoters of eight CIMP-specific promoters (*CACNA1G, CDKN2A, CRABP1, IGF2, MLH1, NEUROG1, RUNX3*, and *SOCS1*), and CIMP-low/negative as 0-5 methylated promoters, as previously described (22, 31). Polymerase chain reaction and pyrosequencing were performed to assess mutations in *KRAS* (codons 12, 13, 61, and 146), *BRAF* (codon 600), and *PIK3CA* (exons 9 and 20) (30, 32, 33).

As previously described (34, 35), the quantitative polymerase chain reaction was conducted to measure the amount of *Fusobacterium nucleatum* (*F. nucleatum*) and *Bifidobacterium genus* DNA in tumor tissue, using SLCO2A1 (for *F. nucleatum*) or a universal 16S primer set (for *Bifidobacterium* genus) as reference genes. We categorized cases with any detectable *F. nucleatum* DNA and *Bifidobacterium* genus as low vs. high based on the median level of *F. nucleatum* and *Bifidobacterium* genus, while other cases were categorized as negative.

### Statistical analyses

All statistical analyses were conducted using SAS software (version 9.4, SAS Institute, Cary, NC, USA). All *P* values were two-sided and we used the stringent α level of 0.005 as recommended by the expert statisticians due to multiple comparisons (36). Our primary hypothesis testing was to determine whether levels of lymphocytic reaction and immune cell densities statistically significantly differed by age at diagnosis (<50 vs. ≥55, 50-54 vs. ≥55). All other assessments, such as assessments of differences in clinical or molecular characteristic by age groups, were secondary analyses. We performed the Chi-squared test or Fisher’s exact test (if appropriate) to compare categorical data between age groups. We performed the Wilcoxon rank-sum test to compare immune cell densities between age groups because immune cell densities were not normally distributed.

## RESULTS

Among 1,518 incident colorectal cancer (CRC) cases in the two cohorts, there were 35 early-onset cases diagnosed at age <50, 73 intermediate-onset cases diagnosed at age 50-54, and 1,410 later-onset cases diagnosed at age ≥55. Table 1 summarizes clinical, pathological, and molecular characteristics according to age at diagnosis.

In our primary hypothesis testing, we assessed whether histopathologic lymphocytic reaction patterns statistically significantly differed by age at diagnosis (Table 2). Although no comparisons showed statistically significant differences at the stringent two-sided α level of 0.005, compared to later-onset CRC, early-onset CRC tended to show lower levels of tumor-infiltrating lymphocytes (P=0.013), intratumoral periglandular reaction (P=0.025), and peritumoral lymphocytic reaction (P=0.044). Considering the link between the gut microbiota and anti-tumor immunity, we investigated differences in *F. nucleatum* or *Bifidobacterium genus* positivity in tumor tissue between age groups (Table 2). However, we did not observe any significance difference. To investigate the potential influence of MSI status on our finding, we also conducted analyses limited to non-MSI-high tumors, which yielded similar patterns to those of the overall analyses although the sample size was limited (Supplementary Table 1).

Furthermore, we assessed whether densities of more specific immune cell subsets (cells/mm^2^) differed by age at diagnosis (Table 3). There were no significant differences in immune cell densities between early-onset CRC and later-onset CRC. Although statistical significance was not reached due to low statistical power, intermediate-onset CRC tended to show lower densities of overall macrophages (median [interquartile range, IQR], 357 [152-670]), M1-like macrophages (45 [18-114]), CD14^+^HLA-DR^+^ cells (mature macrophages) (479 [233-967]), and CD3^+^CD4^+^FOXP3^+^ cells (regulatory T cells) (0 [0-2.0]) compared to later-onset CRC (453 [254-811], P=0.050; 91 [31-211], P=0.062; 730 [377-1239], P=0.015; 0.9 [0-8.5], P=0.039, respectively). We observed similar findings in analyses limited to non-MSI-high tumors (Supplementary Table 2). Detailed data on four age groups (<50, 50-54, 55-69, ≥70) and immune cell densities in tumor intraepithelial and stromal regions are shown in Supplementary Tables 3 and 4.

## DISCUSSION

Colorectal cancer (CRC) is a heterogenous group of neoplastic diseases influenced by tumor-host interactions in the tumor microenvironment (1–3). Thus, comprehensive immunologic analyses of tumor tissue in CRC can shed light on the carcinogenesis process (27, 37–40). In this study, we comprehensively evaluated immune cell infiltrates in CRC by age at diagnosis. The age of 50 years has been used as a cutoff to define early-onset CRC in the field. This cutoff might be at least in part derived from the recommended age to start screening for CRC in the past. However, it remains uncertain whether there is any reasonable cutoff point or there is an “age continuum” in a true biological sense. Especially, it remains to be studied whether CRCs diagnosed at or shortly after age 50 are similar to early-onset CRCs. To address this, we examined immune cell infiltrates in CRC tissue by three age groups (<50, 50-54, ≥55). Although no comparisons showed statistically significant differences at the stringent two-sided α level of 0.005, several potentially interesting results were observed. Compared to later-onset cases diagnosed at age ≥55, early-onset CRC tended to show lower levels of tumor-infiltrating lymphocytes, intratumoral periglandular reaction, and peritumoral lymphocytic reaction. Compared to later-onset cases, intermediate-onset cases tended to show lower densities of overall macrophages, M1-like macrophages, and CD3^+^CD4^+^FOXP3^+^ cells. Though our findings need to be replicated, this study generates several novel hypotheses for further investigation.

Previous studies showed that early-onset CRC was associated with certain pathological and molecular characteristics, including poor tumor differentiation, signet ring cell morphology, and tumor hypomethylation of long interspersed nucleotide element-1 (12, 15–20, 41, 42). However, only few studies have investigated lymphocytic reaction patterns in early-onset CRC (17, 20). One study reported that tumor infiltrating lymphocytes were less common in CRCs diagnosed age <40 compared to those diagnosed at age ≥40 (20). Another study reported that CRCs diagnosed at age ≤40 less commonly showed Crohn’s-like lymphoid reaction compared to cases diagnosed at age >40, whereas there was no significant difference in tumor infiltrating lymphocytes between the two age groups (17). Although statistical power was limited in CRCs before age 55, our data suggest that lymphocytic reactions may be less pronounced in early-onset cases. Lymphocytic reaction patterns reflect the anti-tumor immune response and have been associated with lower CRC mortality (24). Therefore, survival outcomes of early-onset CRC may be at least partly adversely affected by lower levels of lymphocytic reaction.

Detailed features of immune cell infiltrates in early-onset CRC and intermediate-onset CRC diagnosed at age 50-54 are currently unknown. To detect detailed immune cell phenotypes, we employed three multiplex immunofluorescence assays that could simultaneously measure the expression levels of multiple protein markers (26–28). For example, the detection of M1-like and M2-like macrophages requires multimarker combinations, as no single marker has appropriate specificity (43). This study suggests possible differences in the densities of overall and M1-like macrophages and regulatory T cells according to the patient age at CRC diagnosis. Considering the established pro-inflammatory role of M1-like macrophages and the anti-inflammatory role of regulatory T cells (43, 44), our findings may suggest that there are differences in the immune microenvironment between intermediate-onset cases and later-onset cases. Studying the immune cell profiles by age at diagnosis can shed light on how CRC emerges and grows in young adults. Therefore, further investigation is warranted to replicate our findings and elucidate the underlying mechanisms.

We recognize limitations of this study. First, the small sample size of early-onset and intermediate-onset CRC cases limited the statistical power and precluded a conclusive interpretation of the results. Second, measurement errors exist in tissue analysis data. Third, not all incident CRC cases in the cohort studies could be included due to lack of available tumor tissue specimens. Nonetheless, our recent studies that controlled for selection bias through use of inverse probability weighting method (45) did show little evidence for the presence of substantial selection bias in our CRC tissue database (24). Fourth, most of the study subjects were non-Hispanic Caucasians. Hence, independent studies on other populations are needed.

This study has notable strengths. First, the molecular pathological epidemiology database with detailed data on tumor molecular and immune characteristics, which was derived from the two U.S.-wide prospective cohort studies, allowed us to conduct comprehensive analyses of lymphocytic reaction patterns and immune cell densities of early-onset, intermediate-onset, and later-onset CRC cases. Second, the study population was recruited from hospitals throughout the U.S., which increases the generalizability of our results. Third, the current study was based on the assessment of immune cell densities by multiplex immunofluorescence, which is a powerful tool to simultaneously detect multiple epitopes in the context of immune cell biology.

In conclusion, this hypothesis-generating study suggests possible differences in histopathologic lymphocytic reaction patterns, macrophages, and regulatory T cells in the tumor microenvironment by age at diagnosis.

## Supplementary Material

1741556_Sup_tab

## Figures and Tables

**Table 1.** Clinical, pathological, and molecular characteristics of colorectal cancer cases according to age at diagnosis

Characteristics^a^	Total No. (n = 1518)	Age at diagnosis				
		<50 (n = 35)	*P* value (<50 vs. ≥55)^b^	50-54 (n = 73)	*P* value (50-54 vs. ≥55)^c^	≥55 (n = 1410)
Sex						
Female (NHS)	853 (56%)	25 (71%)	0.054	52 (71%)	0.0066	776 (55%)
Male (HPFS)	665 (44%)	10 (29%)		21 (29%)		634 (54%)
Family history of colorectal cancer in a first-degree relative						
Absent	1215 (81%)	27 (77%)	0.62	59 (83%)	0.59	1129 (81%)
Present	293 (19%)	8 (23%)		12 (17%)		273 (19%)
Tumor location						
Proximal colon	733 (48%)	6 (17%)	0.0020	31 (42%)	0.25	696 (46%)
Distal colon	447 (30%)	17 (49%)		24 (33%)		446 (32%)
Rectum	332 (22%)	12 (34%)		18 (25%)		302 (22%)
pT stage						
pT1 (submucosa)	156 (11%)	4 (13%)	0.70	3 (4.4%)	0.22	149 (12%)
pT2 (muscularis propria)	289 (21%)	8 (25%)		14 (21%)		267 (21%)
pT3 (subserosa)	848 (62%)	17 (53%)		45 (66%)		786 (633%)
pT4 (serosa or other organs)	80 (5.8%)	3 (9.4%)		6 (8.3%)		71 (5.6%)
pN stage						
pN0 (0)	848 (64%)	18 (58%)	0.15	30 (48%)	0.036	800 (64%)
pN1 (1-3)	294 (22%)	5 (16%)		19 (31%)		270 (22%)
pN2 (≥4)	192 (14%)	8 (26%)		13 (21%)		171(14%)
AJCC disease stage						
I	358 (26%)	10 (30%)	0.71	11 (17%)	0.034	337 (26%)
II	441 (32%)	8 (24%)		16 (24%)		417 (33%)
III	385 (28%)	11 (33%)		26 (39%)		348 (27%)
IV	190 (14%)	4 (12%)		13 (20%)		173 (14%)
Tumor differentiation						
Well to moderate	1350 (90%)	31 (91%)	0.99	65 (89%)	0.87	1254 (90%)
Poor	156 (10%)	3 (9.0%)		8 (11%)		145 (10%)
MSI status						
Non-MSI-high	1105 (84%)	27 (100%)	0.015	55 (89%)	0.22	1023 (83%)
MSI-high	220 (16%)	0 (0%)		7 (11%)		213 (17%)
CIMP status						
Low/negative	1050 (82%)	28 (97%)	0.029	61 (94%)	0.0090	961 (81%)
High	231 (18%)	1 (3.5%)		4 (6.2%)		226 (19%)
*KRAS* mutation						
Wild-type	733 (58%)	17 (63%)	0.57	41 (66%)	0.18	675 (57%)
Mutant	531 (42%)	10 (37%)		21 (34%)		500 (43%)
*BRAF* mutation						
Wild-type	1132 (85%)	25 (93%)	0.29	58 (89%)	0.27	1049 (84%)
Mutant	206 (15%)	2 (7.4%)		7 (11%)		197 (16%)
*PIK3CA* mutation						
Wild-type	1044 (84%)	23 (85%)	0.99	49 (86%)	0.66	972 (84%)
Mutant	200 (16%)	4 (15%)		8 (14%)		188 (16%)

a Percentage (%) indicates the proportion of cases with a specific clinical or pathological characteristic in all cases according to age categories.

b To compare categorical data between age groups (<50 vs. ≥55), the chi-square test or Fisher’s exact test (if appropriate) was performed.

c To compare categorical data between age groups (50-54 vs. ≥55), the chi-square test or Fisher’s exact test (if appropriate) was performed.

Abbreviations: AJCC, American Joint Committee on Cancer; CIMP, CpG island methylator phenotype; HPFS, Health Professionals Follow-up Study; MSI, microsatellite instability; NHS, Nurses’ Health Study.

**Table 2.** Lymphocytic reaction patterns and microbial features according to age at diagnosis

Characteristics^a^	Total No. (n = 1518)	Age at diagnosis				
		<50 (n = 35)	*P* value (<50 vs. ≥55)^b^	50-54 (n = 73)	*P* value (50-54 vs. ≥55)^c^	≥55 (n = 1410)
Tumor-infiltrating lymphocytes						
Absent/low	1125 (74%)	33 (94%)	0.013	53 (74%)	0.78	1039 (74%)
Intermediate	237 (16%)	2 (5.7%)		13 (18%)		222 (16%)
High	152 (10%)	0 (0%)		6 (8.3%)		146 (10%)
Intratumoral periglandular reaction						
Absent/low	200 (13%)	5 (14%)	0.025	6 (8.2%)	0.20	189 (13%)
Intermediate	1112 (73%)	30 (86%)		60 (82%)		1022 (73%)
High	202 (13%)	0 (0%)		7 (9.6%)		195 (14%)
Peritumoral lymphocytic reaction						
Absent/low	215 (14%)	5 (14%)	0.044	7 (10%)	0.043	203 (15%)
Intermediate	1048 (70%)	29 (83%)		60 (82%)		959 (69%)
High	245 (16%)	1 (2.9%)		6 (8.2%)		238 (17%)
Crohn’s-like lymphoid reaction						
Absent/low	936 (75%)	27 (93%)	0.078	39 (80%)	0.88	870 (75%)
Intermediate	215 (17%)	2 (6.9%)		7 (14%)		206 (18%)
High	92 (7.4%)	0 (0%)		3 (6.2%)		89 (7.6%)
Amount of *F. nucleatum* DNA						
Negative	1083 (87%)	23 (92%)	0.77	52 (90%)	0.85	1008 (87%)
Low	80 (6.4%)	1 (4.0%)		3 (5.2%)		76 (6.6%)
High	77 (6.2%)	1 (4.0%)		3 (5.2%)		73 (6.3%)
Amount of *Bifidobacterium* genus DNA						
Negative	917 (70%)	19 (73%)	0.91	39 (61%)	0.16	860 (71%)
Low	190 (15%)	4 (15%)		14 (22%)		172 (14%)
High	189 (15%)	3 (12%)		11 (17%)		175 (15%)

a Percentage (%) indicates the proportion of cases with a specific clinical or pathological characteristic in all cases according to age categories.

b To compare categorical data between age groups (<50 vs. ≥55), the Fisher’s exact test was performed.

c To compare categorical data between age groups (50-54 vs. ≥55), the chi-square test or Fisher’s exact test (if appropriate) was performed.

**Table 3.** Immune cell densities of colorectal cancer cases according to age at diagnosis

Immune cell densities (cells/mm^2^)^a^	Total No. (n = 966)	Age at diagnosis				
		<50 (n = 19)	*P* value (<50 vs. ≥55)^b^	50-54 (n = 46)	*P* value (50-54 vs. ≥55)^c^	≥55 (n = 875)
**T cells**						
CD3^+^ cells	78 (18-252)	132 (42-189)	0.41	86 (11-251)	0.78	76 (18-255)
CD3^+^CD4^+^ cells	35 (3.4-151)	48 (21-122)	0.60	37 (1.6-107)	0.66	35 (3.4-154)
CD3^+^CD4^+^FOXP3^+^ cells	0.7 (0-8.2)	1.6 (0-6.7)	0.61	0 (0-2.0)	0.039	0.9 (0-8.5)
CD3^+^CD4^+^CD45RO^+^ cells	27 (2.7-119)	41 (17-122)	0.58	29 (0-81)	0.44	25 (2.7-121)
CD3^+^CD4^+^CD45RO^−^ cells	3.5 (0-22)	5.6 (0-19)	0.73	4.1 (0-28)	0.56	3.4 (0-22)
CD3^+^CD8^+^ cells	6.8 (0-30)	7.5 (1.5-24)	0.97	8.0 (0-27)	0.71	6.7 (0-31)
CD3^+^CD8^+^CD45RO^+^ cells	4.4 (0-23)	3.9 (0-9.2)	0.54	3.7 (0-22)	0.53	4.5 (0-23)
CD3^+^CD8^+^CD45RO^−^ cells	1.3 (0-5.5)	2.5 (0-11)	0.10	1.6 (0-9.6)	0.33	1.3 (0-5.2)
CD3^+^CD45RO^+^ cells	43 (5.4-158)	57 (26-128)	0.70	43 (2.9-120)	0.48	43 (5.5-161)
**Macrophages**						
Overall macrophages	449 (250-806)	530 (248-852)	0.91	357 (152-670)	0.050	453 (254-811)
M1-like macrophages	88 (30-210)	65 (33-314)	0.63	45 (18-114)	0.062	91 (31-211)
M2-like macrophages	107 (42-231)	86 (38-209)	0.58	91 (27-219)	0.43	109 (42-233)
**Other myeloid cells**						
CD14^+^ cells	994 (609-1580)	1135 (482-1400)	0.60	812 (473-1352)	0.13	997 (613-1589)
CD14^+^HLA-DR^+^ cells	720 (366-1221)	768 (369-1038)	0.88	479 (233-967)	0.015	730 (377-1239)
CD14^+^HLA-DR^−^ cells	232 (119-391)	202 (108-328)	0.21	274 (107-451)	0.31	228 (119-395)
CD15^+^ cells	97 (28-256)	103 (14-527)	0.73	81 (24-226)	0.48	98 (29-258)
CD15^+^ARG1^+^ cells	79 (21-233)	93 (11-505)	0.78	73 (22-177)	0.49	80 (21-235)
CD15^+^ARG1^−^ cells	11 (3.2-24)	6.1 (2.4-22)	0.47	9.7 (2.4-23)	0.80	11 (3.3-24)
CD15^+^CD33^+^ cells	1.6 (0-11)	4.3 (0-18)	0.31	2.5 (0-15)	0.64	1.5 (0-11)
CD15^+^CD33^−^ cells	89 (26-239)	90 (14-454)	0.74	78 (24-210)	0.50	90 (28-570)

a Each continuous variable is shown as median (IQR).

b To compare continuous variables between age groups (<50 vs. ≥55), the Wilcoxon rank-sum test was performed.

c To compare continuous variables between age groups (50-54 vs. ≥55), the Wilcoxon rank-sum test was performed.

## Abbreviations:

AJCC: American Joint Committee on Cancer
CIMP: CpG island methylator phenotype
CRC: colorectal cancer
FFPE: formalin-fixed paraffin-embedded
HPFS: Health Professionals Follow-up Study
IQR: interquartile range
MSI: microsatellite instability
NHS: Nurses’ Health Study
