# Supplementary material for: Immune cell profiles in the tumor microenvironment of early-onset, intermediate-onset, and later-onset colorectal cancer
Source: Cancer Immunol Immunother. Author manuscript; Available in PMC 2023 Apr 1. (PMC8924022; doi:10.1007/s00262-021-03056-6)
Supplement: 1741556_Sup_tab [file NIHMS1741556-supplement-1741556_Sup_tab.pdf]

Supplementary Table 1. Lymphocytic reaction patterns according to age at diagnosis in non-MSI-high tumors

Characteristics[a]	Total No. (n = 1,103)	Age at diagnosis <50 (n = 27)	*P* value (<50 vs. ≥55)[b]	50-54 (n = 55)	*P* value (50-54 vs. ≥55)[c]	≥55 (n = 1,021)
Tumor-infiltrating lymphocytes						
Absent/low	906 (82%)	25 (93%)	0.46	41 (75%)	0.26	840 (83%)
Intermediate	144 (13%)	2 (7.4%)		11 (20%)		131 (13%)
High	53 (4.8%)	0 (0%)		3 (5.5%)		50 (4.9%)
Intratumoral periglandular reaction						
Absent/low	149 (14%)	4 (15%)	0.29	5 (9.1%)	0.24	140 (14%)
Intermediate	862 (75%)	23 (85%)		48 (87%)		791 (77%)
High	93 (8.4%)	0 (0%)		2 (3.6%)		91 (8.7%)
Peritumoral lymphocytic reaction						
Absent/low	167 (16%)	4 (15%)	0.53	4 (7.3%)	0.063	159 (16%)
Intermediate	810 (74%)	22 (81%)		48 (87%)		740 (73%)
High	123 (11%)	1 (3.7%)		3 (5.5%)		119 (12%)
Crohn's-like lymphoid reaction						
Absent/low	738 (82%)	21 (95%)	0.40	32 (78%)	0.65	685 (82%)
Intermediate	133 (15%)	1 (4.6%)		7 (17%)		125 (15%)
High	32 (3.5%)	0 (0%)		2 (4.9%)		30 (3.6%)

[a] Percentage (%) indicates the proportion of cases with a specific clinical or pathological characteristic according to age categories.
[b] To compare categorical data between age groups (<50 vs. ≥55), the Fisher's exact test was performed.
[c] To compare categorical data between age groups (50-54 vs. ≥55), the Fisher's exact test was performed.

Supplementary Table 2. Immune cell densities of colorectal cancer cases according to age at diagnosis in non-MSI-high tumors

Immune cell densities (cells/mm²)[a]	Total No. (n = 778)	Age at diagnosis <50 (n = 19)	*P* value (<50 vs. ≥55)[b]	50-54 (n = 38)	*P* value (50-54 vs. ≥55)[c]	≥55 (n = 721)
**T cells**						
$CD3^+$ cells	73 (17-256)	132 (42-188)	0.29	90 (15-264)	0.98	69 (16-228)
$CD3^+CD4^+$ cells	36 (3.1-149)	48 (21-122)	0.58	42 (1.8-113)	0.76	35 (3.1-151)
$CD3^+CD4^+FOXP3^+$ cells	0 (0-7.4)	1.6 (0-6.7)	0.51	0 (0-1.7)	0.087	0 (0-7.9)
$CD3^+CD4^+CD45RO^+$ cells	27 (2.6-118)	41 (17-122)	0.54	29 (0-85)	0.51	26 (2.6-119)
$CD3^+CD4^+CD45RO^-$ cells	3.6 (0-21)	5.6 (0-19)	0.71	4.2 (0-29)	0.50	3.4 (0-21)
$CD3^+CD8^+$ cells	5.5 (0-24)	7.5 (1.5-24)	0.75	5.9 (0-31)	0.96	5.5 (0-24)
$CD3^+CD8^+CD45RO^+$ cells	3.6 (0-17)	3.9 (0-9.2)	0.79	2.9 (0-22)	0.78	3.6 (0-17)
$CD3^+CD8^+CD45RO^-$ cells	1.2 (0-4.9)	2.5 (0-11)	0.056	1.7 (0-9.1)	0.22	1.2 (0-4.8)
$CD3^+CD45RO^+$ cells	38 (5.1-150)	57 (26-128)	0.57	37 (3.0-134)	0.60	37 (5.1-158)
**Macrophages**						
Overall macrophages	403 (232-732)	530 (248-852)	0.56	423 (146-670)	0.30	398 (236-733)
M1-like macrophages	79 (28-186)	65 (33-314)	0.90	45 (18-114)	0.11	81 (28-186)
M2-like macrophages	99 (42-226)	86 (38-209)	0.71	91 (20-219)	0.37	101 (42-227)
**Other myeloid cells**						
$CD14^+$ cells	953 (570-1490)	1135 (482-1400)	0.81	812 (479-1289)	0.22	955 (571-1509)
$CD14^+HLA\text{-}DR^+$ cells	678 (360-1168)	768 (369-1038)	0.89	517 (234-968)	0.11	682 (363-1170)
$CD14^+HLA\text{-}DR^-$ cells	224 (115-384)	202 (108-328)	0.27	274 (105-436)	0.40	223 (116-386)
$CD15^+$ cells	76 (23-221)	103 (14-527)	0.96	75 (23-226)	0.66	76 (23-218)
$CD15^+ARG1^+$ cells	63 (18-197)	93 (11-505)	0.89	61 (19-176)	0.73	63 (17-197)
$CD15^+ARG1^-$ cells	9.1 (3.0-21)	6.1 (2.4-22)	0.63	9.2 (2.3-22)	0.77	9.4 (3.1-21)
$CD15^+CD33^+$ cells	1.4 (0-9.1)	4.3 (0-18)	0.18	2.5 (0-14)	0.32	1.3 (0-8.9)
$CD15^+CD33^-$ cells	71 (21-207)	90 (14-454)	0.95	72 (23-205)	0.66	71 (22-207)

[a] Each continuous variables is shown as median (IQR).
[b] To compare continuous variables between age groups (<50 vs. ≥55), the Wilcoxon rank-sum test was performed.
[c] To compare continuous variables between age groups (50-54 vs. ≥55), the Wilcoxon rank-sum test was performed.

Supplementary Table 3. Clinical, pathological, and molecular characteristics of colorectal cancer cases according to age at diagnosis (<50, 50-54, 55-69, ≥70)

Characteristics[a]	Total No. (n = 1518)	Age at diagnosis <50 (n = 35)	50-54 (n = 73)	55-69 (n = 671)	≥70 (n = 739)	*P* value across 4 groups[b]
Sex						0.0046
Female (NHS)	853 (56%)	25 (71%)	52 (71%)	418 (62%)	358 (48%)	
Male (HPFS)	665 (44%)	10 (29%)	21 (29%)	253 (38%)	381 (52%)	
Family history of colorectal cancer in a first-degree relative						0.76
Absent	1215 (81%)	27 (77%)	59 (83%)	542 (82%)	587 (80%)	
Present	293 (19%)	8 (23%)	12 (17%)	125 (19%)	148 (20%)	
Tumor location						0.0048
Proximal colon	733 (48%)	6 (17%)	31 (42%)	284 (43%)	408 (56%)	
Distal colon	447 (30%)	17 (49%)	24 (33%)	219 (33%)	187 (25%)	
Rectum	332 (22%)	12 (34%)	18 (25%)	163 (24%)	139 (19%)	
pT stage						0.45
pT1 (submucosa)	156 (11%)	4 (13%)	3 (4.4%)	79 (13%)	70 (11%)	
pT2 (muscularis propria)	289 (21%)	8 (25%)	14 (21%)	112 (18%)	155 (23%)	
pT3 (subserosa)	848 (62%)	17 (53%)	45 (66%)	386 (63%)	400 (61%)	
pT4 (serosa or other organs)	80 (5.8%)	3 (9.4%)	6 (8.3%)	35 (5.7%)	36 (5.5%)	
pN stage						0.038
pN0 (0)	848 (64%)	18 (58%)	30 (48%)	378 (63%)	442 (66%)	
pN1 (1-3)	294 (22%)	5 (16%)	19 (31%)	134 (22%)	136 (21%)	
pN2 (≥4)	192 (14%)	8 (26%)	13 (21%)	85 (14%)	86 (13%)	
AJCC disease stage						0.13
I	358 (26%)	10 (30%)	11 (17%)	154 (25%)	183 (28%)	
II	441 (32%)	8 (24%)	16 (24%)	199 (32%)	218 (33%)	
III	385 (28%)	11 (33%)	26 (39%)	174 (28%)	174 (26%)	
IV	190 (14%)	4 (12%)	13 (20%)	91 (15%)	82 (12%)	
Tumor differentiation						0.94
Well to moderate	1350 (90%)	31 (91%)	65 (89%)	600 (90%)	654 (89%)	
Poor	156 (10%)	3 (9.0%)	8 (11%)	65 (9.8%)	80 (11%)	

Tumor-infiltrating lymphocytes						0.027
Absent/low	1125 (74%)	33 (94%)	53 (74%)	512 (76%)	527 (71%)	
Intermediate	237 (16%)	2 (5.7%)	13 (18%)	91 (14%)	131 (18%)	
High	152 (10%)	0 (0%)	6 (8.3%)	66 (9.9%)	80 (11%)	
Intratumoral periglandular reaction						0.0009
Absent/low	200 (13%)	5 (14%)	6 (8.2%)	75 (11%)	114 (15%)	
Intermediate	1112 (73%)	30 (86%)	60 (82%)	516 (77%)	506 (69%)	
High	202 (13%)	0 (0%)	7 (9.6%)	79 (12%)	116 (16%)	
Peritumoral lymphocytic reaction						<0.0001
Absent/low	215 (14%)	5 (14%)	7 (10%)	77 (11%)	126 (17%)	
Intermediate	1048 (70%)	29 (83%)	60 (82%)	510 (77%)	449 (61%)	
High	245 (16%)	1 (2.9%)	6 (8.2%)	79 (12%)	159 (22%)	
Crohn’s-like lymphoid reaction						0.40
Absent/low	936 (75%)	27 (93%)	39 (80%)	413 (76%)	457 (74%)	
Intermediate	215 (17%)	2 (6.9%)	7 (14%)	94 (17%)	112 (18%)	
High	92 (7.4%)	0 (0%)	3 (6.2%)	38 (7.0%)	51 (8.2%)	
MSI status						0.030
Non-MSI-high	1105 (84%)	27 (100%)	55 (89%)	523 (87%)	500 (79%)	
MSI-high	220 (16%)	0 (0%)	7 (11%)	80 (13%)	133 (21%)	
CIMP status						0.0037
Low/negative	1050 (82%)	28 (97%)	61 (94%)	504 (86%)	457 (76%)	
High	231 (18%)	1 (3.5%)	4 (6.2%)	82 (14%)	144 (18%)	
KRAS mutation						0.35
Wild-type	733 (58%)	17 (63%)	41 (66%)	347 (59%)	328 (56%)	
Mutant	531 (42%)	10 (37%)	21 (34%)	245 (41%)	255 (44%)	
BRAF mutation						0.28
Wild-type	1132 (85%)	25 (93%)	58 (89%)	524 (86%)	525 (82%)	
Mutant	206 (15%)	2 (7.4%)	7 (11%)	84 (14%)	113 (18%)	
PIK3CA mutation						0.89
Wild-type	1044 (84%)	23 (85%)	49 (86%)	464 (84%)	508 (84%)	
Mutant	200 (16%)	4 (15%)	8 (14%)	88 (16%)	100 (16%)	

Amount of *F. nucleatum* DNA						0.92
Negative	1083 (87%)	23 (92%)	52 (90%)	489 (88%)	519 (86%)	
Low	80 (6.4%)	1 (4.0%)	3 (5.2%)	31 (5.6%)	45 (7.5%)	
High	77 (6.2%)	1 (4.0%)	3 (5.2%)	33 (6.0%)	40(6.6%)	
Amount of *Bifidobacterium* genus DNA						
Negative	917 (70%)	19 (73%)	39 (61%)	414 (72%)	445 (71%)	0.68
Low	190 (15%)	4 (15%)	14 (22%)	83 (14%)	89 (14%)	
High	189 (15%)	3 (12%)	11 (17%)	82 (14%)	93 (15%)	

[a] Percentage (%) indicates the proportion of cases with a specific clinical or pathological characteristic in cases according to age categories.
[b] To compare categorical data across four age groups (<50, 50-54, 55-69, ≥70), the chi-square test or Fisher's exact test (if appropriate) was performed.
Abbreviations: AJCC, American Joint Committee on Cancer; CIMP, CpG island methylator phenotype; HPFS, Health Professionals Follow-up Study; MSI, microsatellite instability; NHS, Nurses' Health Study.

Supplementary Table 4. Immune cell densities of colorectal cancer cases according to age at diagnosis (<50, 50-54, 55-69, ≥70)

Immune cell densities (cells/mm^2)[a]	Total No. (n = 966)	Age at diagnosis <50 (n = 19)	50-54 (n = 46)	55-69 (n = 452)	≥70 (n = 449)	*P* value across 4 groups[b]
**Tumor intraepithelial region**						
CD3$^+$ cells	35 (9.0-116)	36 (11-85)	29 (4.1-92)	33 (8.0-112)	36 (11-122)	0.53
CD3$^+$CD4$^+$ cells	8.5 (0-47)	6.5 (1.6-20)	6.1 (0-25)	7.7 (0-49)	9.6 (0-48)	0.81
CD3$^+$CD4$^+$FOXP3$^+$ cells	0 (0-2.1)	0 (0-2.8)	0 (0-0)	0 (0-2.0)	0 (0-2.8)	0.12
CD3$^+$CD4$^+$CD45RO$^+$ cells	7.1 (0-39)	5.4 (0-14)	2.2 (0-21)	6.2 (0-39)	8.1 (0-41)	0.49
CD3$^+$CD4$^+$CD45RO$^-$ cells	0 (0-5.3)	0.9 (0-5.0)	0 (0-4.5)	0 (0-5.6)	0 (0-5.5)	0.79
CD3$^+$CD8$^+$ cells	3.1 (0-18)	4.9 (0-11)	2.0 (0-19)	3.0 (0-57)	3.4 (0-18)	0.88
CD3$^+$CD8$^+$CD45RO$^+$ cells	1.7 (0-13)	2.5 (0-6.8)	1.3 (0-12)	1.4 (0-12)	2.2 (0-14)	0.74
CD3$^+$CD8$^+$CD45RO$^-$ cells	0 (0-3.1)	2.1 (0-4.8)	0 (0-3.4)	0 (0-3.2)	0 (0-2.9)	0.23
CD3$^+$CD45RO$^+$ cells	13 (0-59)	7.4 (4.3-40)	12 (0-49)	13 (0-52)	14 (2.0-69)	0.56
Overall macrophage	163 (79-323)	192 (73-323)	128 (43-292)	143 (71-299)	187 (99-360)	0.19
M1-like macrophage	26 (7.3-69)	78 (3.1-78)	33 (2.1-33)	23 (6.0-62)	32 (9.5-84)	0.065
M2-like macrophage	46 (15-108)	24 (16-74)	37 (7.1-111)	43 (14-100)	52 (18-118)	0.44
CD14$^+$ cells	146 (69-303)	152 (64-271)	126 (53-302)	142 (63-294)	151 (72-331)	0.58
CD14$^+$HLA-DR+ cells	97 (38-221)	118 (33-236)	63 (27-173)	99 (35-222)	99 (43-224)	0.36
CD14$^+$HLA-DR- cells	30 (10-67)	33 (10-57)	36 (11-101)	28 (10-68)	32 (10-67)	0.72
CD15$^+$ cells	31 (6.7-105)	25 (5.0-289)	36 (0-101)	26 (5.0-95)	35 (8.5-113)	0.20
CD15$^+$ARG1$^+$ cells	26 (4.4-97)	25 (4.9-289)	27 (0-96)	22 (2.8-84)	32 (6.0-104)	0.13
CD15$^+$ARG1$^-$ cells	0 (0-7.6)	2.0 (0-8.8)	0 (0-7.1)	0 (0-6.9)	1.7 (0-7.9)	0.68
CD15$^+$CD33$^+$ cells	0 (0-0)	0 (0-1.4)	0 (0-3.2)	0 (0-3.7)	0 (0-3.7)	0.99
CD15$^+$CD33$^-$ cells	28 (6.6-98)	24 (5.0-289)	34 (0-87)	25 (5.0-90)	32 (8.3-104)	0.21
**Tumor stromal region**						
CD3$^+$ cells	147 (22-499)	240 (118-558)	226 (17-587)	144 (21-517)	134 (22-453)	0.68
CD3$^+$CD4$^+$ cells	77 (3.8-362)	79 (47-261)	109 (3.7-436)	72 (3.3-376)	84 (4.1-354)	0.97
CD3$^+$CD4$^+$FOXP3$^+$ cells	0 (0-22)	3.9 (0-19)	0 (0-11)	0 (0-19)	0 (0-25)	0.43
CD3$^+$CD4$^+$CD45RO$^+$ cells	58 (0-290)	72 (31-235)	66 (0-284)	50 (0-307)	63 (2.3-289)	0.96
CD3$^+$CD4$^+$CD45RO$^-$ cells	7.2 (0-48)	11 (0-29)	12 (0-102)	7.3 (0-51)	5.6 (0-46)	0.23
CD3$^+$CD8$^+$ cells	10 (0-54)	12 (0-41)	8.3 (0-56)	9.0 (0-52)	11 (0-59)	0.66
CD3$^+$CD8$^+$CD45RO$^+$ cells	4.8 (0-41)	0 (0-18)	0 (0-31)	4.0 (0-40)	6.7 (0-44)	0.11
CD3$^+$CD8$^+$CD45RO$^-$ cells	0 (0-10)	7.7 (0-16)	2.2 (0-22)	0 (0-11)	0 (0-8.3)	0.12
CD3$^+$CD45RO$^+$ cells	86 (6.2-351)	109 (56-235)	74 (0-351)	81 (4.6-343)	87 (7.8-365)	0.91
Overall macrophage	866 (486-1413)	920 (612-1541)	689 (239-1431)	841 (424-1341)	894 (549-1463)	0.15
M1-like macrophage	170 (61-382)	126 (39-509)	96 (24-222)	157 (57-378)	206 (75-402)	0.017
M2-like macrophage	193 (69-428)	192 (64-327)	142 (66-382)	192 (66-409)	208 (78-470)	0.64
CD14$^+$ cells	1824 (1199-2843)	1812 (1271-3039)	1641 (944-2429)	1789 (1177-2667)	1879 (1234-3039)	0.13
CD14$^+$HLA-DR+ cells	1336 (741-2261)	1869 (882-2096)	989 (406-2028)	1252 (766-2135)	1422 (780-2462)	0.034
CD14$^+$HLA-DR- cells	425 (255-662)	389 (198-618)	520 (277-843)	414 (254-663)	425 (256-656)	0.23
CD15$^+$ cells	164 (51-430)	99 (47-610)	130 (49-357)	146 (42-406)	191 (64-444)	0.20
CD15$^+$ARG1$^+$ cells	130 (37-368)	95 (28-561)	107 (34-324)	116 (31-354)	142 (47-388)	0.21
CD15$^+$ARG1$^-$ cells	19 (5.5-43)	6.9 (4.1-48)	18 (4.1-41)	16 (4.9-43)	21 (6.9-43)	0.19

CD15$^+$CD33$^+$ cells	1.9 (0-17)	5.3 (0-38)	3.5 (0-21)	0 (0-13)	2.1 (0-20)	0.36
CD15$^+$CD33$^-$ cells	156 (12-387)	96 (17-561)	126 (45-331)	135 (42-364)	180 (61-412)	0.19
**All tumor region**						
CD3$^+$ cells	78 (18-252)	132 (42-189)	86 (11-251)	71 (17-255)	82 (19-254)	0.68
CD3$^+$CD4$^+$ cells	35 (3.4-151)	48 (21-122)	37 (1.6-107)	34 (3.0-154)	35 (3.7-154)	0.89
CD3$^+$CD4$^+$FOXP3$^+$ cells	0.7 (0-8.2)	1.6 (0-6.7)	0 (0-2.0)	0.6 (0-7.1)	1.2 (0-10)	0.14
CD3$^+$CD4$^+$CD45RO$^+$ cells	27 (2.7-119)	41 (17-122)	29 (0-81)	25 (2.6-119)	27 (3.2-128)	0.73
CD3$^+$CD4$^+$CD45RO$^-$ cells	3.5 (0-22)	5.6 (0-19)	4.1 (0-28)	3.2 (0-22)	3.6 (0-22)	0.82
CD3$^+$CD8$^+$ cells	6.8 (0-30)	7.5 (1.5-24)	8.0 (0-27)	5.8 (0-29)	7.2 (1.2-35)	0.45
CD3$^+$CD8$^+$CD45RO$^+$ cells	4.4 (0-23)	3.9 (0-9.2)	3.7 (0-22)	3.8 (0-19)	5.3 (0-27)	0.22
CD3$^+$CD8$^+$CD45RO$^-$ cells	1.3 (0-5.5)	2.5 (0-11)	1.6 (0-9.6)	1.3 (0-6.2)	1.3 (0-4.9)	0.29
CD3$^+$CD45RO$^+$ cells	43 (5.4-158)	57 (26-128)	43 (2.9-120)	38 (4.5-150)	46 (6.7-171)	0.63
Overall macrophage	449 (250-806)	530 (248-852)	357 (152-670)	424 (222-786)	477 (292-828)	0.15
M1-like macrophage	88 (30-210)	65 (33-314)	45 (18-114)	79 (28-199)	106 (34-235)	0.022
M2-like macrophage	107 (42-231)	86 (38-209)	91 (27-219)	109 (41-231)	115 (50-233)	0.63
CD14$^+$ cells	994 (609-1580)	1135 (482-1400)	812 (473-1352)	964 (583-1509)	1031 (624-1671)	0.18
CD14$^+$HLA-DR+ cells	720 (366-1221)	768 (369-1038)	479 (233-967)	366 (366-1173)	767 (389-1302)	0.041
CD14$^+$HLA-DR- cells	232 (119-391)	202 (108-328)	274 (107-451)	240 (118-407)	221 (125-384)	0.45
CD15$^+$ cells	97 (28-256)	103 (14-527)	81 (24-226)	79 (24-248)	116 (36-275)	0.13
CD15$^+$ARG1$^+$ cells	79 (21-233)	93 (11-505)	73 (22-177)	65 (17-217)	92 (28-246)	0.13
CD15$^+$ARG1$^-$ cells	11 (3.2-24)	6.1 (2.4-22)	9.7 (2.4-23)	8.8 (2.9-23)	12 (3.9-26)	0.17
CD15$^+$CD33$^+$ cells	1.6 (0-11)	4.3 (0-18)	2.5 (0-15)	1.3 (0-10)	1.8 (0-12)	0.51
CD15$^+$CD33$^-$ cells	89 (26-239)	90 (14-454)	78 (24-210)	74 (23-232)	109 (35-259)	0.12

[a] Each continuous variable is shown as median (IQR).
[b] To compare continuous variables across four age groups (<50, 50-54, 55-69, ≥70), the Kruskal-Wallis test was performed.
